# Conservation Agriculture Practices in Rainfed Uplands of India Improve Maize-Based System Productivity and Profitability

**DOI:** 10.3389/fpls.2016.01008

**Published:** 2016-07-15

**Authors:** Aliza Pradhan, Travis Idol, Pravat K. Roul

**Affiliations:** ^1^Department of Natural Resources and Environmental Management, University of Hawaii at Manoa, HonoluluHI, USA; ^2^Directorate of Planning, Monitoring and Evaluation, Orissa University of Agriculture and TechnologyBhubaneswar, India

**Keywords:** rainfed uplands, system productivity, maize equivalent yield, dominance analysis

## Abstract

Traditional agriculture in rainfed uplands of India has been experiencing low agricultural productivity as the lands suffer from poor soil fertility, susceptibility to water erosion and other external pressures of development and climate change. A shift toward more sustainable cropping systems such as conservation agriculture production systems (CAPSs) may help in maintaining soil quality as well as improving crop production and farmer’s net economic benefit. This research assessed the effects over 3 years (2011–2014) of reduced tillage, intercropping, and cover cropping practices customized for maize-based production systems in upland areas of Odisha, India. The study focused on crop yield, system productivity and profitability through maize equivalent yield and dominance analysis. Results showed that maize grain yield did not differ significantly over time or among CAPS treatments while cowpea yield was considered as an additional yield in intercropping systems. Mustard and horsegram grown in plots after maize cowpea intercropping recorded higher grain yields of 25 and 37%, respectively, as compared to those without intercropping. Overall, the full CAPS implementation, i.e., minimum tillage, maize–cowpea intercropping and mustard residue retention had significantly higher system productivity and net benefits than traditional farmer practices, i.e., conventional tillage, sole maize cropping, and no mustard residue retention. The dominance analysis demonstrated increasing benefits of combining conservation practices that exceeded thresholds for farmer adoption. Given the use of familiar crops and technologies and the magnitude of yield and income improvements, these types of CAPS should be acceptable and attractive for smallholder farmers in the area. This in turn should support a move toward sustainable intensification of crop production to meet future household income and nutritional needs.

## Introduction

Traditional, rainfed agro-ecosystems are still important in India, contributing up to 44% of the country’s annual food production. In Odisha, India, one of the poorest states in the country, maize-based cropping systems are common in the interior districts, which are dominated by tribal communities (Pradhan et al., 2015). A common cropping system is maize (*Zea mays* L.) followed by mustard (*Brassica juncea* L.) and then a fallow period during the dry season. During the onset of the monsoonal rainy season, seeds of open-pollinated and low-yielding varieties of maize are broadcast sown into fields prepared by multiple plowings with a simple bullock-drawn plow that cuts into the soil but does not turn it over like a moldboard plow. Uncomposted farmyard manure and low levels of urea (∼10 kg ha^-1^) are typically the only soil amendments provided for the crops. After harvest, if residual soil moisture is sufficient, farmers will plow the field again and broadcast sow seeds of local varieties of mustard.

While maize stover is typically left in fields after harvest, it is not deliberately utilized for mulch or soil cover. Plowing for mustard tends to incorporate most of the residue, leaving little soil cover. For mustard, the entire aboveground stem is harvested and the seeds removed by threshing for extraction of oil. Residues from threshing are typically piled and burned as waste. During the dry season that follows mustard harvesting, livestock are generally allowed to freely graze crop fields, eating any remaining live or dead plant material.

This combination of using traditional crop varieties, multiple plowings, repeated maize cultivation, no attempt at soil cover, and low inputs has resulted in low yields and thus low food security and income for farmers in these districts. One approach to addressing these issues is the introduction and adaptation of conservation agriculture production systems (CAPSs). CAPS are defined as integrated production systems consisting of minimum soil disturbance, appropriate crop rotation or intercropping, and continuous organic soil cover (Roul et al., 2015). The integrated nature of CAPS builds on decades of research in more large-scale and mechanized farming systems in which zero- or minimum-tillage systems were developed and combined with crop rotation and residue retention or cover cropping to reduce soil erosion and related declines in soil and natural resource quality (Idol, 2015). Only more recently have these concepts been adapted and applied to smallholder cropping systems, where conventional Green Revolution approaches to improving crop yield (better seed, higher input rates, mechanization) are unfeasible or have been unsustainable (Giller et al., 2009; Gilbert, 2012).

Given the obvious variability of agro-ecological environments, cropping systems, and farmer capacities and preferences, there is not a single CAPS that applies worldwide. Therefore, successful introduction of CAPS depends upon adapting and tailoring the basic principles to the local context. As maize is the staple crop in tribal areas of Odisha, a maize-based CAPS is needed to improve agronomic, environmental, and socioeconomic sustainability in these areas. Therefore, the objective of this study was to investigate the effects of maize-based CAPS on crop yield, system productivity, and profitability in a rainfed low-input region of Odisha, India.

## Materials and Methods

### Experimental Site

**FIGURE 1:**
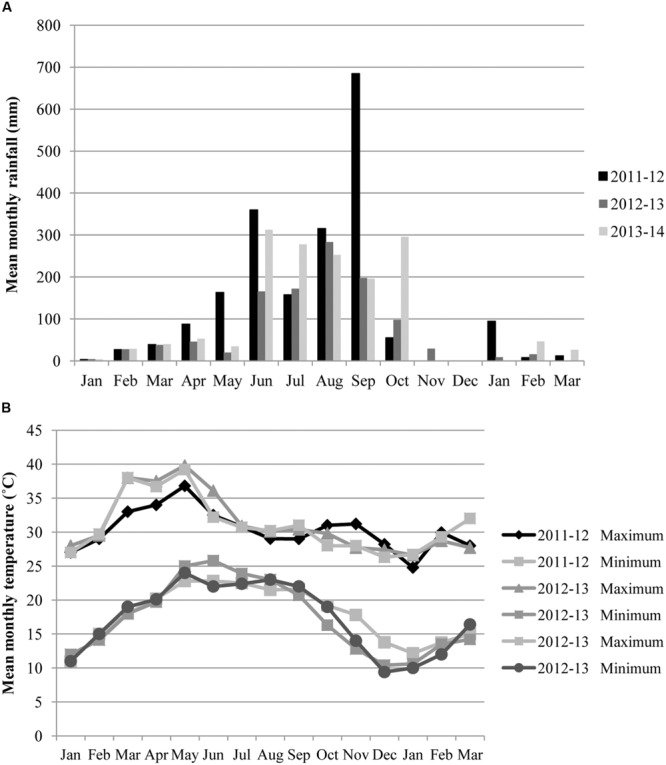
**Mean monthly rainfall **(A)** and mean monthly maximum and minimum temperature **(B)** during cropping periods of 2011–12, 2012–13, and 2013–14**.

A field experiment was conducted in rainfed uplands at the Regional Research and Technology Transfer Station (RRTTS; 85° 34′ 30.61″ E, 20° 50′ 55.38″ N; 499 m above mean sea level) of Orissa University of Agriculture and Technology (OUAT) in the Keonjhar district, Odisha, India over three cropping cycles, from 2011 to 2014. The soil of the study site is mainly developed from colluvial-alluvial deposits in piedmont plain with soil texture ranging from sandy clay loam to sandy loam with pH (6.5) and classified as Fluventic Haplustepts (Inceptisol). The basic soil characteristics of the experimental site for (0–20) cm soil depth, measured just before laying out the field in 2011–12 were; total carbon 1.86%, total nitrogen (N) 0.2%, available phosphorous (P) 15.8 kg ha^-1^ (Olsen’s P) and available potassium (K) 341.8 kg ha^-1^ (ammonium acetate flame photometry method). The climate of the study area is sub-humid tropical with average annual rainfall of 1500 mm, with more than 75% of the rainfall received in the months from May to September (**Figure 1**). The usual cropping system of the study site is maize during the rainy season (mid June–September) followed by mustard (*Brassica campestris* L.) as a post-rainy season crop (October–January).

### Selection of CAPS Treatments

A set of CAPSs practices were selected based on discussions with farmers, researchers, and extension personnel regarding their tillage and crop preference, past cropping history of the area, market demand, other threats and challenges (Lai et al., 2012). In order to reduce soil erosion, a minimum tillage method of plowing once before planting was proposed as an alternative to the conventional practice of plowing three times. Again, because of the central importance of maize as a staple food and the limitation of land for rotation, an intercropping rather than crop rotation option was selected. Cowpea (*Vigna unguiculata* L.) was considered suitable as an intercrop as it is a legume and will help in biological nitrogen fixation; has a high market value (twice that of maize); and local farmers have had some previous experience with growing and selling it. In order to address the cover crop and residue management principle, horse gram (*Macrotyloma uniflorum*) was selected as an alternative post-rainy season monocrop in addition to mustard. Horse gram was the preferred cover crop option as it provides economic yield as well as acts as a legume soil cover. Both horse gram and mustard grow reasonably well on residual soil moisture and mature better with dry weather during the late vegetative and reproductive stages (i.e., during January).

### Experimental Design and Layout

**FIGURE 2:**
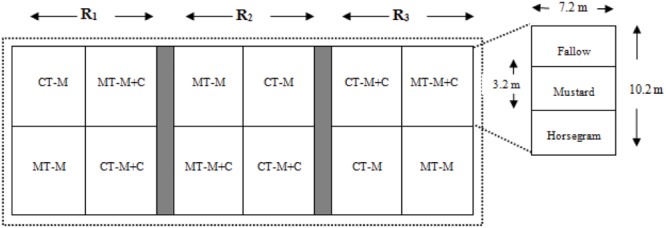
**Layout of the experimental plots under CAPS management regime.** (CT-M: conventional tillage- maize cropping; CT-M+C: conventional tillage-maize+cowpea; MT-M: minimum tillage- maize cropping; MT-M+C: minimum tillage-maize+cowpea; R_1_, R_2_, R_3_: represent replications of the experiment).

The experiment was initiated in May 2011. The experimental design was a randomized complete block, split-plot design with three replicates, one per block (**Figure 2**). Each plot dimension was 10.2 m in length × 7.2 m in width. Tillage and cropping system were the main plot treatments, and cover crop selection was the split-plot treatment. The main plot treatments compared four management practices: conventional tillage with maize cropping (CT-M); conventional tillage with maize+cowpea (1:1; *Vigna unguiculata* L.; CT-M+C); minimum tillage with maize cropping (MT-M); and minimum tillage with maize+cowpea (1:1; MT-M+C). Conventional tillage consisted of plowing the field with a bullock-drawn plow. A single pass of the plow was done during the pre-monsoonal rains, a few days to weeks prior to the expected heavy monsoonal rains. After the onset of monsoonal rains, the field was criss-cross plowed. Though farmers’ normal practice is to broadcast maize seed throughout the plot, this study used line sowing of maize seed to maintain consistency with the minimum tillage treatment. Minimum tillage consisted of a single plowing prior to sowing followed by strip-tilling rows with hand-held hoes to sow maize seed. Hand-weeding with hoes occurred several times afterward in both the tillage systems. No novel tools or equipment often used with conservation agriculture, e.g., seed drills or chisel plows, were used due to lack of local availability and farmer experience with them. After harvest of maize and cowpea, the plot was prepared for planting of cover crops. Each plot was split into thirds and randomly assigned to one of three cover crop treatments: no cover crop, i.e., fallow (F), mustard (*Brassica campestris* L.) as cover crop (Mu), and horse gram (*Macrotyloma uniflorum*)as cover crop (H).

### Selection of Crop Varieties and Calendar of Agricultural Operations

The crop varieties and spacing used in the study are shown in **Table 1** Maize was harvested manually approximately 90 days after sowing. Because cowpea is an indeterminately flowering and fruiting crop, manual harvesting of mature seed pods began approximately 40 days after sowing and continued until 60 days.

**Table 1:** Details of the crop varieties, spacing (cm) and plant population (plants ha^**-1**^).

Crop	Selected variety	Spacing (cm)	Plant population (plants ha^-1^)
Maize	Nilesh	60 × 30	55, 555
Cowpea	Hariyalli Bush	30 × 15	222, 222
Mustard	Parvati	30 × 10	333, 333
Horsegram	Athagada Local	30 × 10	333, 333

After final harvest of both maize and cowpea, crop residues were left as such in the fields, and the plot was prepared by strip-tilling rows with hand held hoes for planting of cover crops (**Figure 2**). The cover crops were harvested approximately 75 days after sowing. After threshing, all residues were collected on plastic tarpaulins and returned back to their respective plots.

### Crop Harvest and Yield Estimation

Yield measurements of maize, mustard, and horsegram grains were done after harvesting and threshing the crops at crop maturity. Grain yield of these crops are reported at 12% grain moisture content. Cowpea green pods were picked manually at 7-days intervals, and their fresh weight after each harvest was recorded. Grain and stover yields of the crops were determined by harvesting three areas in a 1 m × 1 m grid cell within each plot. To estimate the effect of CAPS on total system productivity, yields of all non-maize crops were converted to maize equivalent yield (MEY) based on market prices using Eq. (1). The market prices were collected from local farmers’ markets during 2011–12, 2012–13, and 2013–14.

(1) Maize equivalent yield (kg ha-1) = Crop yield (kg ha-1) × Crop price ($kg-1)/Maize Price ($kg-1)               

### Economic Analysis

Economic performance of the systems was assessed using the CIMMYT economic training manual (CIMMYT, 1988), which included step-wise procedures of partial budgeting, dominance, and marginal analyses. The partial budgeting used total variable cost, gross field benefits and net field benefits under each scenario. The variable costs included human labor, bullock drawn plow used for land preparation, and cost of inputs such as seed, fertilizer, and farm yard manure (FYM). The unit of human labor was based on labor day(s) ha^-1^ and was calculated by recording the time required for each agricultural activity and converting them to labor days (8 h being equivalent to 1 labor day). The cost of labor was calculated using the minimum wage rate for the study years as per the Labor Law of the Government of India. Similarly, the time required by the bullock drawn plow to complete the tillage practice was recorded and expressed as day(s) ha^-1^ (8 h being equivalent to 1 day). Gross field benefits were calculated by multiplying the field price of maize by the MEY, where field price of maize was estimated by taking the price that farmers receive for the crop when they sell it, and subtracting all the associated costs associated with harvest and sale proportional to the yield. Net benefits were calculated as the difference between gross field benefits and total variable costs. Next, a dominance analysis was carried out by first listing the treatments in order of increasing variable costs. Then, any treatment having net benefits less than or equal to that of a treatment of lower variable costs was considered dominated. In order to have a firm treatment recommendation, a marginal analysis was done using marginal rate of return and a net benefit curve. Marginal rate of return was calculated by marginal net benefit (i.e., change in net benefits) divided by marginal cost (i.e., change in total variable costs), expressed as a percentage. A plot displaying net benefits against total variable cost was created to represent the net benefits curve. It is assumed that farmers will continue to invest as long as the returns to each extra unit invested (measured by the marginal rate of return) are higher than the cost of the extra unit invested (measured by the minimum acceptable rate of return). Minimum acceptable return is the level of additional returns, beyond the cost of capital that will satisfy the farmers that their investment is worthwhile. For the reduced tillage treatment, we used an accepted level of 80% (CIMMYT, 1988). Finally, a sensitivity analysis was performed to evaluate the stability of recommendations against price fluctuations.

### Data Analysis

After ensuring normality and homogeneity of variance of the data, they were subjected to repeated measures multivariate analysis of variance (MANOVA) and analyzed for significance using appropriate *F*-test (SAS Institute, 2001). Where the *F*-test were significant, means were compared using Tukey’s honest significance difference (HSD) test at *P* < 0.05. Due to non-significant effect of year, pooled data over the three cropping seasons were taken into consideration for analysis of crop yield and system productivity. Economic analysis of crop production though did not include any statistical analysis but was based on the statistical outcome of total system productivity.

## Results and Discussion

### Effect of Conservation Agriculture Production Systems (CAPSs) on Maize and Cowpea Yields

**FIGURE 3:**
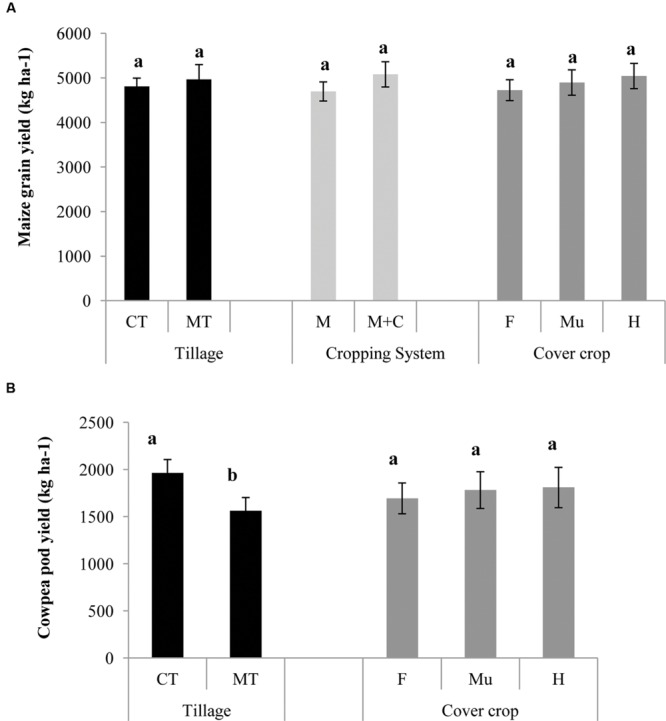
**Effect of conservation agriculture production systems (CAPSs) on **(A)** maize and **(B)** cowpea yields in kg ha^-1^, averaged over three cropping seasons (2011–2014).** Bars represent mean value ±1 standard error. Within each CAPS component, means followed by the same letter are not significantly different at *P* < 0.05 according to Tukey’s HSD test (*n* = 3). CT, conventional tillage; MT, minimum tillage; M, only maize cropping; M+C, maize cowpea intercropping; F, fallow (no cover crop); Mu, mustard residue as cover; H, horsegram residue as cover.

Maize yields in all treatments and years averaged 4888 kg ha^-1^ and did not differ statistically by treatment or year (**Figure 3A**). This is considerably greater than the national average of 2285 kg ha^-1^ (Directorate of Maize Research, India, 2011–12). This might be attributed to favorable rainfall distribution pattern and soil fertility of the site coupled with the use of an improved maize variety and application of recommended agronomic practices. In most cases, results from long term conservation agriculture studies have shown that maize yields in the initial years are not significantly different from conventional practices (Thierfelder et al., 2013). Immediate yield benefits of CA were observed only in some field studies such as in Ngwira et al. (2012) where benefits of conservation agriculture on maize yields were realized in the very first year itself in one of the study sites. There are also numerous other studies regarding the variability of short term yield responses (positive, neutral, or negative yield responses) to conservation agriculture practices (Lal, 1986; Gill and Aulakh, 1990; Mbagwu, 1990; Mupangwa et al., 2012). In general, conservation agriculture yield benefits took longer to establish a clear upward trend. The reason is generally attributed to the time necessary to build soil fertility and to adapt to the new conservation agriculture system – a phenomenon called “age hardening” for soils transitioning from intensive tillage to minimum or no-tillage (Dexter et al., 1988). Even though short-term yield effects of conservation agriculture are variable over space and time, yield responses over a longer time period tend to be neutral to positive (Giller et al., 2009; Gilbert, 2012).

Cowpea did not appear to compete with the maize crop, as there was no significant difference in maize yields between maize monocrop and maize–cowpea intercrop (**Figure 3B**). Past studies have also shown a full range of responses of maize to intercropping, including yield reductions (Adeniyan et al., 2007; Lemlem, 2013), neutral responses (Watiki et al., 1993; Thobatsi, 2009; Ngwira et al., 2012) and yield increases (Nzabi et al., 2000; Mpairwe et al., 2002; Dapaah et al., 2003). In our study, the neutral response might be due to the delayed sowing of cowpea, intended to minimize competition with maize during the critical crop establishment stage (Thierfelder et al., 2012). Thus, cowpea yield may be considered as an additional yield in intercropping systems. As there is a good market for the crop in the region, getting a ‘bonus’ yield from such areas of existing land constraints will not only improve household income but also improves food and nutritional security. Furthermore, such diversification of maize with cowpea can reduce the risk of complete crop failure in times of drought as was reported by Rusinamhodzi et al. (2012). Tillage did have a significant effect on both cowpea pod and stover yield (*P* < 0.05). Yield of cowpea pods and stover were 26 and 30% greater respectively, in conventional than minimum tillage (**Figure 3B**). Deeper plowing in conventional tillage might have facilitated better root growth of cowpea and thereby showing increased yield. In the long term, increased production and retention of legume biomass may improve the short-term system performance by controlling runoff and by stimulating macrofauna activity (Mannering and Meyer, 1963; Lal, 1988; Mando et al., 1999). It should also lead to increased water infiltration from the creation of a larger number of root channels (Baudron et al., 2012). Cowpea yields in 2012–13 and 2013–14 were significantly higher than in 2011–12. An unusually heavy rainfall of around 685 mm during September might have damaged the cowpea crops leading to lower yield in 2011–12 (**Figure 1A**).

### Effect of Conservation Agriculture Production Systems (CAPSs) on Mustard and Horsegram Yields

**FIGURE 4:**
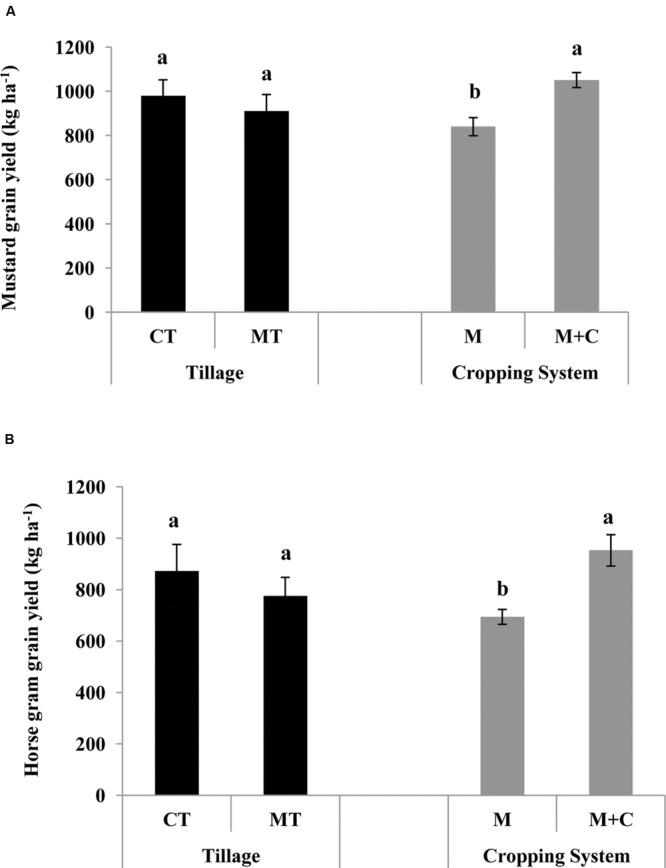
**Effect of CAPSs on **(A)** mustard and **(B)** horsegram yields in kg ha^-1^, averaged over three cropping seasons (2011–2014).** Bars represent mean value ±1 standard error. Within each CAPS component, means followed by the same letter are not significantly different at *P* < 0.05 according to Tukey’s HSD test (*n* = 3). CT, conventional tillage; MT, minimum tillage; M, only maize cropping; M+C, maize cowpea intercropping; F, fallow (no cover crop); Mu, mustard residue as cover; H, horsegram residue as cover.

While the cover crop treatments did not affect maize or cowpea yields, there was a significant effect of cropping system on both mustard and horsegram yields (**Figures 4A,B**). Mustard and horsegram grown in plots after maize+cowpea intercropping had higher grain yields of 25 and 37%, respectively, as compared to those without intercropping. This might be due to more soil nitrogen through biological nitrogen fixation in intercropped plots.

While cover crops can improve soil quality and thus long-term system productivity, for many smallholders in seasonally dry areas, crop residue is an important source of livestock fodder (Mtambanengwe and Mapfumo, 2005; Giller et al., 2009; Umar et al., 2011; Valbuena et al., 2012). Moreover, fields left fallow during this period are traditionally available for communal grazing (McDowell, 1988; Shepherd, 1992). An advantage of mustard over horsegram is that the mustard stover is generally avoided by livestock, reducing the risk of loss when returned and applied as surface mulch. This infers better acceptability of mustard residue retention over horsegram as communal grazing plays an important role in small holder farming systems.

### Effect of Conservation Agriculture Production Systems (CAPS) on Total System Productivity

**FIGURE 5:**
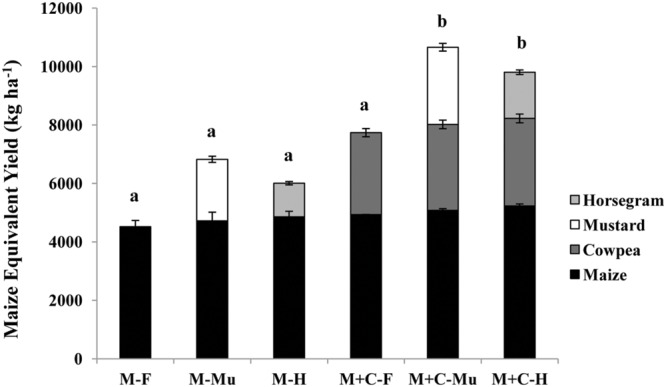
**Effect of CAPSs (cropping ^∗^ cover cropping) on total system productivity in terms of maize equivalent yield (MEY) in kg ha^-1^, averaged over three cropping seasons (2011–2014).** Bars represent mean value ±1 standard error. Within each CAPS component, means followed by the same letter are not significantly different at *P* < 0.05 according to Tukey’s HSD test (*n* = 3). M, only maize cropping; M+C, maize cowpea intercropping; F, fallow (no cover crop); Mu, mustard residue as cover; H, horsegram residue as cover.

Total system productivity over the initial 3 years of crop management was estimated by analyzing the yield contributions of cowpea, mustard, and horse gram toward MEY under different CAPS (**Figure 5**). Both intercropping and cover cropping significantly increased total system productivity, due not only to the additional yield but also their higher market price, 1.5–2.0 times that of maize. Similarly, intercropping plus cover cropping performed significantly better than intercropping followed by fallow, but there was no difference between cover crops (mustard or horsegram). As there was no effect of tillage, similar system productivity can be achieved with less labor.

N.B: the market price of maize grain $0.17 kg^-1^, cowpea pod $0.28 kg^-1^, mustard seed $0.43 kg^-1^, and horsegram seed $0.28 kg^-1^ [1 US Dollar (USD) = 58 Indian National Rupee (INR)]; Price of crops was collected from local market survey.

### Economic Analysis of Conservation Agriculture Production Systems (CAPSs)

The economic analysis for all the crops individually and on a system basis was done considering all the variable costs, gross field benefits and net benefits (**Table 2** and **3**). The analysis was based on pooled data over 3 years. Conventional tillage with intercropping followed by cultivation of mustard had highest total variable costs of $604 ha^-1^. This was mainly due to an additional inputs (seeds + fertilizer) and labor for growing the additional crops. The lowest variable costs were under conventional tillage with maize cropping followed by no cover crop ($370 ha^-1^). Minimum tillage overall was only slightly lower in variable cost than conventional tillage (∼2%). The reduced labor for plowing and land preparation under reduced tillage was largely offset by the increased labor requirement for weeding. This has also been reported in previous studies (Giller et al., 2009; Mazvimavi and Twomlow, 2009). Cowpea intercropping, however, reduced weeding as it formed a closed canopy in the maize inter-row space. This is in agreement with Olsen et al. (2005) who reported that formation of a closed canopy through legume intercropping helps in controlling weeds. Similarly, according to Banik et al. (2006), density and biomass of weeds in diversified cropping systems diminished significantly when compared with single culturing of each component of the diversified system.

**Table 2:** Total variable cost incurred for different conservation agricultural production systems (CAPS).

Agricultural operations		CT-M		CT-M+C		MT-M		MT-M+C		Mustard		Horsegram	
	Price per unit ($)	No. of units	Total cost ($ ha^-1^)	No. of units	Total cost ($ ha^-1^)	No. of units	Total cost ($ ha^-1^)	No. of units	Total cost ($ ha^-1^)	No. of units	Total cost ($ ha^-1^)	No. of units	Total cost ($ ha^-1^)
(a) Land preparation													
• Bullock plowing [day(s)]	2.59	5	12.95	5	12.95	2	5.18	2	5.18				
• Labor day(s)	2.59	25	64.75	25	64.75	15	38.85	15	38.85	10	25.9	10	25.9
(b) Sowing													
• Seed													
Maize	2.24	15	33.6	15	33.6	15	33.6	15	33.6				
Cowpea	7.59			10	75.9			10	75.9				
Mustard	0.79									7.5	5.925		
Horsegram	0.34											25	8.5
• Farm yard manure (FYM)	10.34	5	51.7	5	51.7	5	51.7	5	51.7				
• Fertilizer													
DAP	0.22	87	19.14	130	28.6	87	19.14	130	28.6	43	9.46	87	19.14
Urea	0.09	140	12.6	145	13.05	140	12.6	145	13.05	70	6.3	9	0.81
MOP	0.11	65	7.15	106	11.66	65	7.15	106	11.66	43	4.73	43	4.73
• Labor day(s)	2.59	10	25.9	15	38.85	10	25.9	15	38.85	10	25.9	10	25.9
(c) Intercultural operations [Labor day(s)]													
• First weeding, top dressing, earthing up	2.59	40	103.6	45	116.55	50	129.5	45	116.55	20	51.8	15	38.85
• Second weeding and top dressing	2.59	15	38.85	10	25.9	25	64.75	10	25.9				
Total variable cost ($ ha^-1^)			370		474		388		440		130		124

*All prices in $ indicate US dollar and 1 US $ = 58 INR (Indian national Rupee, average of 2011–2014).*

**Table 3:** Partial budget and dominance analysis of conservation agriculture production systems (CAPSs).

Tillage	Cropping system	Residue cover	^a^Average yield (kg ha^-1^)	^b^Adjusted yield (kg ha^-1^)	^c^Gross field benefits ($ ha^-1^)	^d^Total variable costs ($ ha^-1^)	^e^Net benefits ($ ha^-1^)
Coventional Tillage	Maize	Fallow	4777	4538	681	370	311
		Mustard	7186	6827	1024	500	524aaa
		Horsegram	6237	5925	889	494	395
	Maize+cowpea	Fallow	7735	7348	1102	474	628
		Mustard	10585	10056	1508	604	904
		Horsegram	9882	9388	1408	598	810
Minimum Tillage	Maize	Fallow	4260	4047	607	388	219
		Mustard	6461	6138	921	518	403
		Horsegram	5776	5487	823	512	311
	Maize+cowpea	Fallow	7739	7352	1103	440	663
		Mustard	10731	10195	1529	570	959
		Horsegram	9726	9239	1386	564	822

*^a^Average yield is pooled data of total system productivity in terms of maize equivalent yield over the years.*

*^b^Adjusted yield is the average yield adjusted downward by a certain percentage (5% in this case) to reflect the difference between the experimental yield and the yield farmers could expect from the same treatment. Downward adjustment by 5% was decided based on the actual yield differences between actual on station and on farm study results.*

*^c^Gross field benefits were calculated by multiplying the field price of maize by the adjusted yield; field price of maize was calculated by taking the price that farmers receive for the crop when they sell it, and subtracting all the associated costs associated with harvest, and sale proportional to the yield.*

*^d^Total variable costs indicate the sum of all the costs that vary for a particular treatment.*

*^e^Net benefits are calculated by subtracting gross field benefits from total variable costs.*

*†Net benefits in italics indicate a treatment that is dominated, i.e., the net benefits are less than or equal to that of a treatment with lower variable costs.*

**FIGURE 6:**
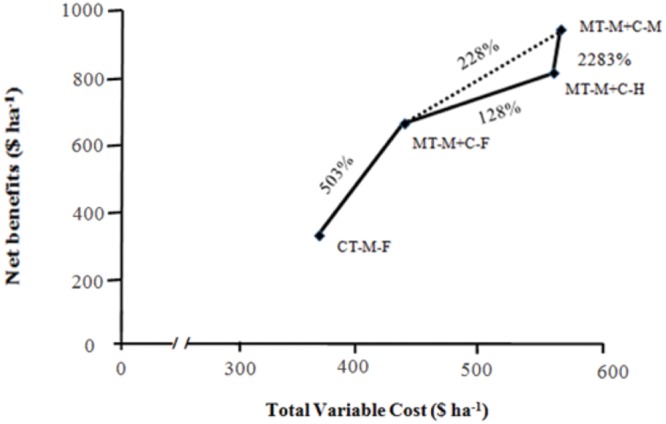
**Marginal analysis of non-dominated CAPS treatments showing net benefit curve and marginal rate of return (%)**.

Both gross and net field benefits were highest under minimum tillage with intercropping followed by mustard (**Table 3**). The lowest gross and net benefits were under minimum tillage with sole maize and no cover crop. The dominance analysis resulted in four treatments that improved net benefits compared to treatments with lower variable cost: CT-M-F and the three MT-M+C treatments. Marginal analysis of all these selected treatments showed a fivefold increase in marginal benefits by shifting from CT-M-F to MT-M+C-F (**Figure 6**). Adding mustard as a cover crop had a higher marginal net benefit (228%) than horsegram (128%). This was primarily due to the higher economic yield of mustard; the increased cost was almost the same. We observed that such attractive marginal rates of return will help in popularizing conservation agriculture among smallholder farmers as monetary gains act as a prime driver for adoption (Erenstein et al., 2008). Further, higher marginal rates of return will enable the farmers to invest in inputs such as seeds and fertilizer. The sensitivity analysis showed that shifting to intercropping and using mustard as a cover crop both met the recommended minimum rate of return (80%), even under an increase in labor costs of 33–40% (**Table 4**). The marginal net benefits of horsegram were close to 80% under these scenarios.

**Table 4:** Sensitivity analysis of CAPS treatments under different unit labor cost scenarios.

^a^Treatments	Unit labor cost ($2.59 day^-1^)			Unit labor cost ($3.44 day^-1^)			Unit labor cost ($3.62 day^-1^)		
	Total variable costs ($ ha^-1^)	Net benefits ($ ha^-1^)	Marginal rate of return (%)	Total variable costs ($ ha^-1^)	Net benefits ($ ha^-1^)	Marginal rate of return (%)	Total variable costs ($ ha^-1^)	Net benefits ($ ha^-1^)	Marginal rate of return (%)
CT-M-F	370	311		447	234		463	218	
MT-M+C-F	440	663	503	512	591	549	527	576	559
MT-M+C-H	564	822	128	666	720	84	687	699	77
MT-M+C-Mu	570	959	2280	676	853	1330	698	831	1200

*^a^Represents the CAPS treatments that remain non-dominated.*

*CT, conventional tillage; MT, minimum tillage; M, only maize cropping; M+C, maize cowpea intercropping; F, fallow (no cover crop); Mu, mustard residue as cover; H, horsegram residue as cover.*

Percentage values along the lines indicate marginal rate of return, which is calculated by marginal net benefit (i.e., change in net benefits) divided by marginal cost (i.e., change in total variable costs), expressed as a percentage.

## Conclusion

Agriculture in developing countries primarily focuses on finding a sustainable agricultural technology that meets the demands of smallholder farmers while maintaining or improving soil fertility. Though there is no universal strategy to end challenges to food security and rural poverty but it was evident from the study that combining and simultaneously applying location-specific and low-input conservation agriculture practices such as minimum tillage, diversified cropping system through maize+cowpea coupled with residue retention of mustard helps in optimizing resource use efficiency and maximize productivity of traditional smallholder farming systems. Given the use of familiar crops and technologies and the significant economic gains of this improved system, it should be acceptable and attractive to smallholder tribal farmers in Eastern India. Further, institutionalizing CAPS into relevant government ministries and departments and regional institutions is required for sustainability of the technology. Local, national and regional policy and decision makers could spearhead and support the formulation and development of strategies and mechanisms for scaling up the technology.

## Author Contributions

AP: conducted the whole research, data compilation and analysis, manuscript preparation; TI: supervised the whole research and manuscript preparation; PR: supervised the research trial at the study site.

## Conflict of Interest Statement

The authors declare that the research was conducted in the absence of any commercial or financial relationships that could be construed as a potential conflict of interest.
